# Lateral parabrachial neurons innervate orexin neurons projecting to brainstem arousal areas in the rat

**DOI:** 10.1038/s41598-019-39063-y

**Published:** 2019-02-26

**Authors:** Yosuke Arima, Shigefumi Yokota, Masashi Fujitani

**Affiliations:** Department of Anatomy and Neuroscience, Shimane University School of Medicine, 89-1 Enya-cho, Izumo-shi, Shimane 693–8501 Japan

## Abstract

Orexin (ORX) neurons in the hypothalamus send their axons to arousal-promoting areas. We have previously shown that glutamatergic neurons in the lateral parabrachial nucleus (LPB) innervate ORX neurons. In this study, we examined potential pathways from the LPB to ORX neurons projecting to arousal-promoting areas in the brainstem by a combination of tract-tracing techniques in male Wistar rats. We injected the anterograde tracer biotinylated dextranamine (BDA) into the LPB and the retrograde tracer cholera toxin B subunit (CTb) into the ventral tegmental area, dorsal raphe nucleus, pedunculopontine tegmental nucleus, laterodorsal tegmental area, or locus coeruleus (LC). We then analyzed the BDA-labeled fibers and ORX-immunoreactive neurons in the hypothalamus. We found that double-labeled ORX and CTb neurons were the most abundant after CTb was injected into the LC. We also observed prominently overlapping distribution of BDA-labeled fibers, arising from neurons located in the lateral-most part of the dorsomedial nucleus and adjacent dorsal perifornical area. In these areas, we confirmed by confocal microscopy that BDA-labeled synaptophysin-immunoreactive axon terminals were in contiguity with cell bodies and dendrites of CTb-labeled ORX-immunoreactive neurons. These results suggest that the LPB innervates arousal-promoting areas via ORX neurons and is likely to promote arousal responses to stimuli.

## Introduction

Orexin (ORX) is a small hypothalamic neuropeptide, synthesized by a cluster of neurons in the lateral hypothalamus. There are two subtypes of ORX neuropeptides; orexin A and orexin B (also known as hypocretin 1 and hypocretin 2)^1^. These peptides play a critical role in the regulation of sleep and wakefulness^2^. ORX neurons are specifically localized to the hypothalamus, including the perifornical area (PeF), lateral hypothalamus (LH), and dorsomedial hypothalamic nucleus (DMH)^1,3^. ORX neurons receive projections from multiple regions of the brain and project to a wide variety of brain regions. Activity of ORX neurons produces arousal^4^, and excitatory inputs to ORX neurons are crucial for maintaining widespread arousal in the brain. In addition, within the ventral tegmental area (VTA), dorsal raphe nucleus (DR), pedunculopontine tegmental nucleus (PPT), laterodorsal tegmental area (LDT), locus coeruleus (LC)^5,6^, monoaminergic neurons in the VTA, DR, and LC and cholinergic neurons in the PPT and LDT have been shown to receive projections from ORX neurons and these neurons are also well known to promote arousal, projecting to widespread brain areas directly or indirectly^7^.

The parabrachial nuclei, a group of nuclei surrounding the superior cerebellar peduncle along its course through the dorsolateral pons, are divided by the peduncle into medial and lateral nuclei. The medial parabrachial nucleus receives projections from the rostral, gustatory portion of the nucleus of the solitary tract. Both nuclei receive projections from the more caudal portion of the nucleus of the solitary tract, where general visceral afferents terminate^8^. The lateral parabrachial nuclei (LPB) receive nociceptive inputs from medullary (trigeminal) and spinal lamina I neurons^9,10^. The LPB constitutes a main relay center for these inputs to areas of the forebrain, including the hypothalamus, amygdala, and bed nucleus of the stria terminalis^11–15^. In addition, The LPB is divided into distinct subnuclei in the rat. Each subnuclei associates with a unique set of afferents and efferents^16^ and the central lateral subnucleus densely project to the hypothalamus^12,14,15^. We previously reported that ORX neurons in the suprafornical area, which is the lateral-most part of the DMH and adjacent dorsal PeF, just dorsal to the fornix of the hypothalamus, receive excitatory vesicular glutamate transporter 2 (VGLUT2) positive LPB fibers, and asymmetrical synapses are formed between these fibers and neurons^17^. A recent study reported that hypercarbia activates LPB neurons, and that disrupted glutamate signaling in the LPB delays arousal induced by hypercarbia in a mouse model of obstructive sleep apnea^18^. In addition, pain, cold, and nausea also excite neurons in the LPB; this pathway may promote arousal in response to a variety of interoceptive stimuli. Furthermore, chemogenetic activation of the parabrachial nucleus induces activation of ORX neurons of the lateral hypothalamus and promotes wakefulness^19^.

Altogether, these data allow us to hypothesize that LPB fibers could form synaptic contacts with ORX neurons projecting to brainstem arousal areas such as the VTA, DR, PPT, LDT and LC. In the present study, we used a combination of tract-tracing techniques to provide novel evidence for the existence of pathways from the LPB to ORX hypothalamic neurons and then to brainstem arousal areas in male Wistar rats.

## Results

### Distribution of ORX-immunoreactive (IR) neurons projecting to the brainstem arousal areas

**Figure 1:**
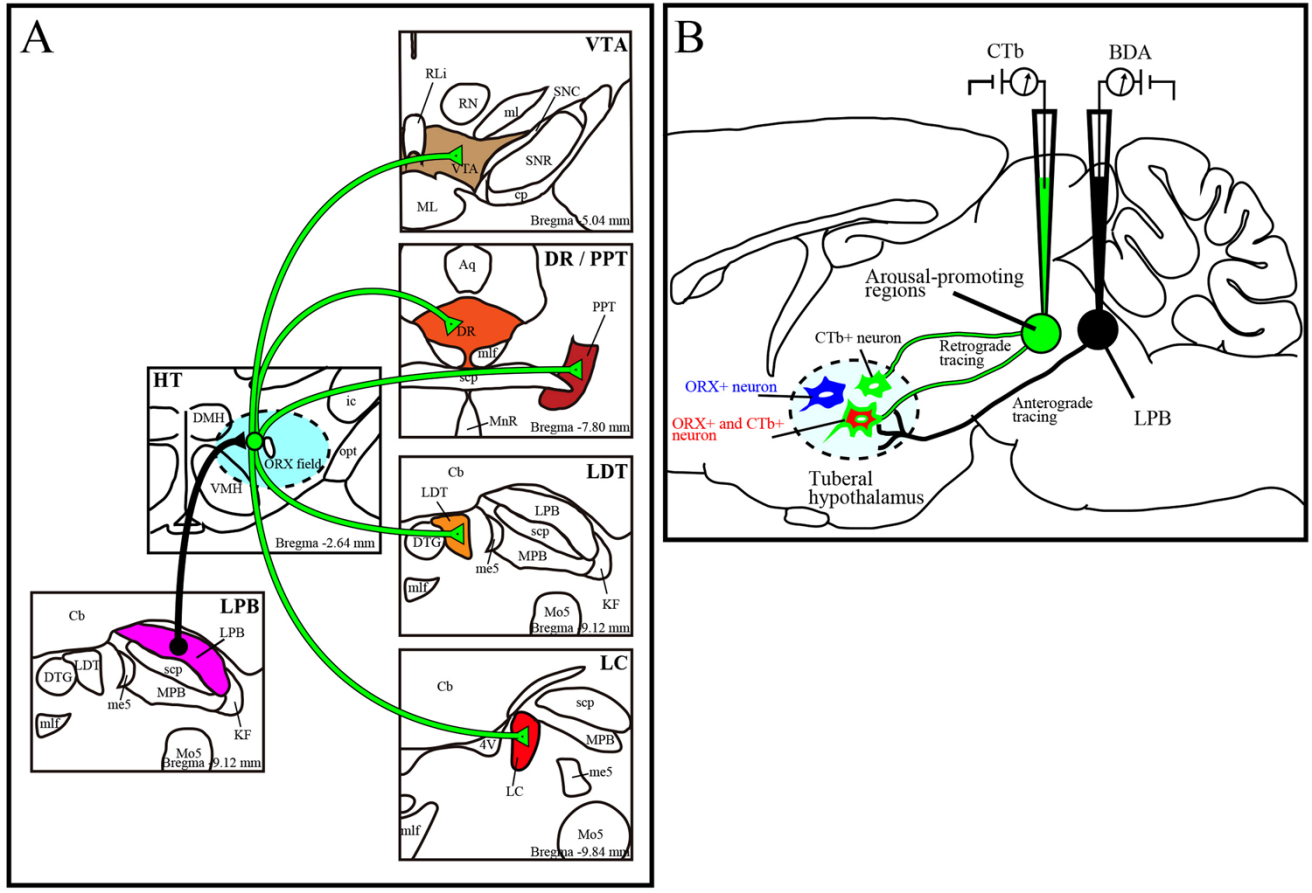
Schematic diagram of this research. (**A**) Diagram of axonal projections from LPB to brainstem arousal areas via ORX neurons which were newly identified in this study. (**B**) Schematic drawings of combined anterograde (black) and retrograde (green) tract-tracing method. Immunohistochemistry was performed on sections of the tuberal hypothalamus (dotted line). CTb negative and ORX positive neurons (ORX+) are represented as blue neurons, CTb positive ORX negative (CTb+) cells are as green neurons and CTb positive ORX positive neurons (ORX+ and CTb+) are as red neurons. LPB; lateral parabrachial nucleus, ORX; orexin, CTb; cholera toxin subunit B, BDA; biotinylated dextranamine, VTA; ventral tegmental area, PPT; pedunculopontine tegmental nucleus, LDT; laterodorsal tegmental area, LC; locus coeruleus, DR; dorsal raphe nucleus.

Since our main interest was arousal-promoting brainstem areas such as the VTA, DR, PPT, LDT and LC, we examined projections from LPB neurons to these areas via the tuberal hypothalamus, as illustrated in Fig. 1A,B. To do this, we injected biotinylated dextranamine (BDA) into the LPB to label neurons projecting to the ORX field in the hypothalamus anterogradely, and also injected cholera toxin subunit B (CTb) into the ipsilateral VTA, DR, PPT, LDT or LC to label neurons from the ORX field retrogradely (Fig. 1A,B). We performed immunohistochemical analysis to detect BDA, CTb and ORX in the lateral hypothalamus (Fig. 1B).

**Figure 2:**
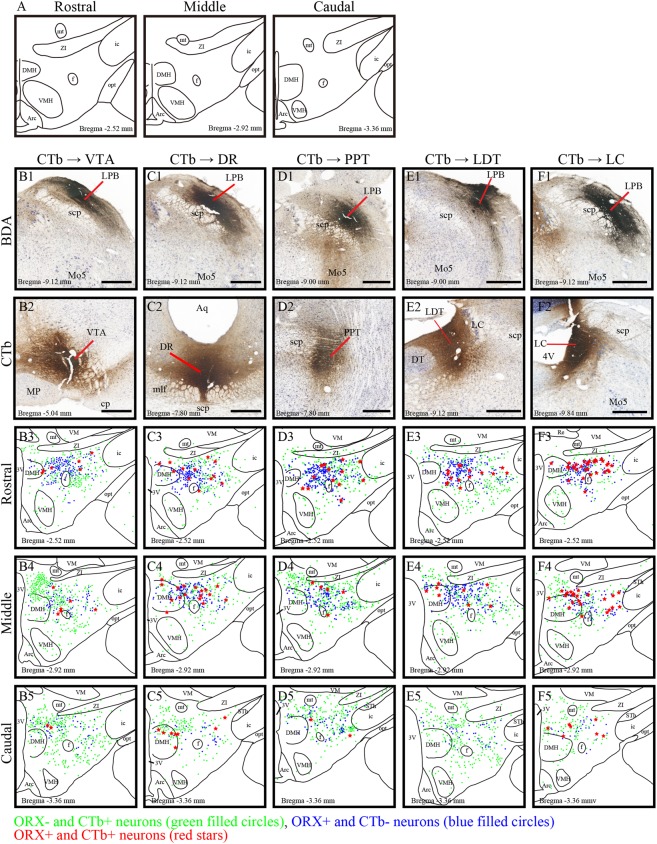
Distribution of ORX-immunoreactive (IR) neurons projecting to the brainstem arousal areas. (**A**) Schematic drawings of three sections which represent the approximate posterior levels to bregma −2.54 mm (rostral), −2.92 mm (middle) and −3.36 mm (Caudal). (**B**) Photomicrographs showing the injection sites of BDA into the LPB (B1-F1) and CTb into the VTA (B2), DR (C2), PPT (D2), LDT (E2) and LC (F2). (B3- F5) Line drawings showing the distribution of CTb-IR neurons (green filled circles), ORX-IR neurons (blue filled circles), and ORX and CT- double-IR neurons (red stars) in the three regions of hypothalamus. The distance (mm) behind the bregma is noted at the bottom. Scale bars, 500 μm. CTb; cholera toxin b subunit; ORX: orexin; IR: immunoreactive; VTA: ventral tegmental area; DR: dorsal raphe; PPT: pedunculopontine nucleus; LDT: laterodorsal tegmental nucleus; LC: locus ceruleus; BDA: biotinylated dextranamine.

First, we observed that ORX-IR neurons were distributed predominantly in the perifornix (PeF) and dorsal LH in addition to the DMH across the tuberal hypothalamus (Supplementary Fig. 1 and Fig. 2B3–F5). We detected more ORX-IR neurons in rostral parts of the lateral hypothalamus, as shown in Supplementary Fig. 1 and in Fig. 2B3–F5.

We then analyzed the distribution of CTb-labeled neurons in the hypothalamus. We first confirmed successful CTb injection into the ipsilateral VTA, DR, PPT, LDT and LC (Fig. 2B2,C2,D2,E2 and F2). We confirmed that these CTb deposits were in the center of neurons immunolabeled with antibodies against tyrosine hydroxylase (TH) in the VTA and LC (Supplementary Figs 3A–C and 7A–C), against 5-hydroxytryptamine (5-HT) in the DR (Supplementary Fig. 4A–C), and against choline acetyltransferase (ChAT) in the PPT and LDT (Supplementary Figs 5A–C and 6A–C).

We then examined CTb-labeled neurons projecting to the VTA, DR, PPT, LDT and LC. They were predominantly distributed in the ipsilateral hypothalamus on the injection side of the VTA, PPT, LDT and LC, 2~5 times more than on the contralateral side (Supplementary Fig. 2). CTb injections in the DR only resulted in the detection of a large number of CTb-labeled neurons bilaterally in the dorsal half of the LH as well as in the dorsomedial nucleus (Supplementary Fig. 2). In addition, we detected CTb-labeled neurons distributed from the preoptic regions to the most caudal portions of the LH (Fig. 2B3–F5). These neurons in the LH and surrounding nuclei were densely organized as a band, and their distribution spanned from the lateral border of the paraventricular hypothalamic nucleus and arched dorsally over the fornix before curving ventrolaterally into the juxtacapsular LH. Moreover, we observed a moderate number of cells in the VMH that showed a sparse distribution in the arcuate nucleus (Fig. 2B3–F5). More specifically, when CTb was injected in the LDT, CTb-labeled neurons were found to be densely localized around the inner boundary of the optic tract (Fig. 2E3–E5). For CTb injection in both the VTA and PPT, large numbers of neurons were densely distributed in the dorsal part of the DMH (Fig. 2B4,B5 and D4).

We observed ORX and CTb double-IR neurons localized from the lateral part of DMH to the medial part of PeF (Fig. 2B3–F5). In particular, when CTb was injected into the LC, the labeled neurons showed dense localization in the lateral part of DMH and ventral part of PeF (Fig. 2F3–F5).

### Abundant innervation of LC by hypothalamic orexin neurons

**Figure 3:**
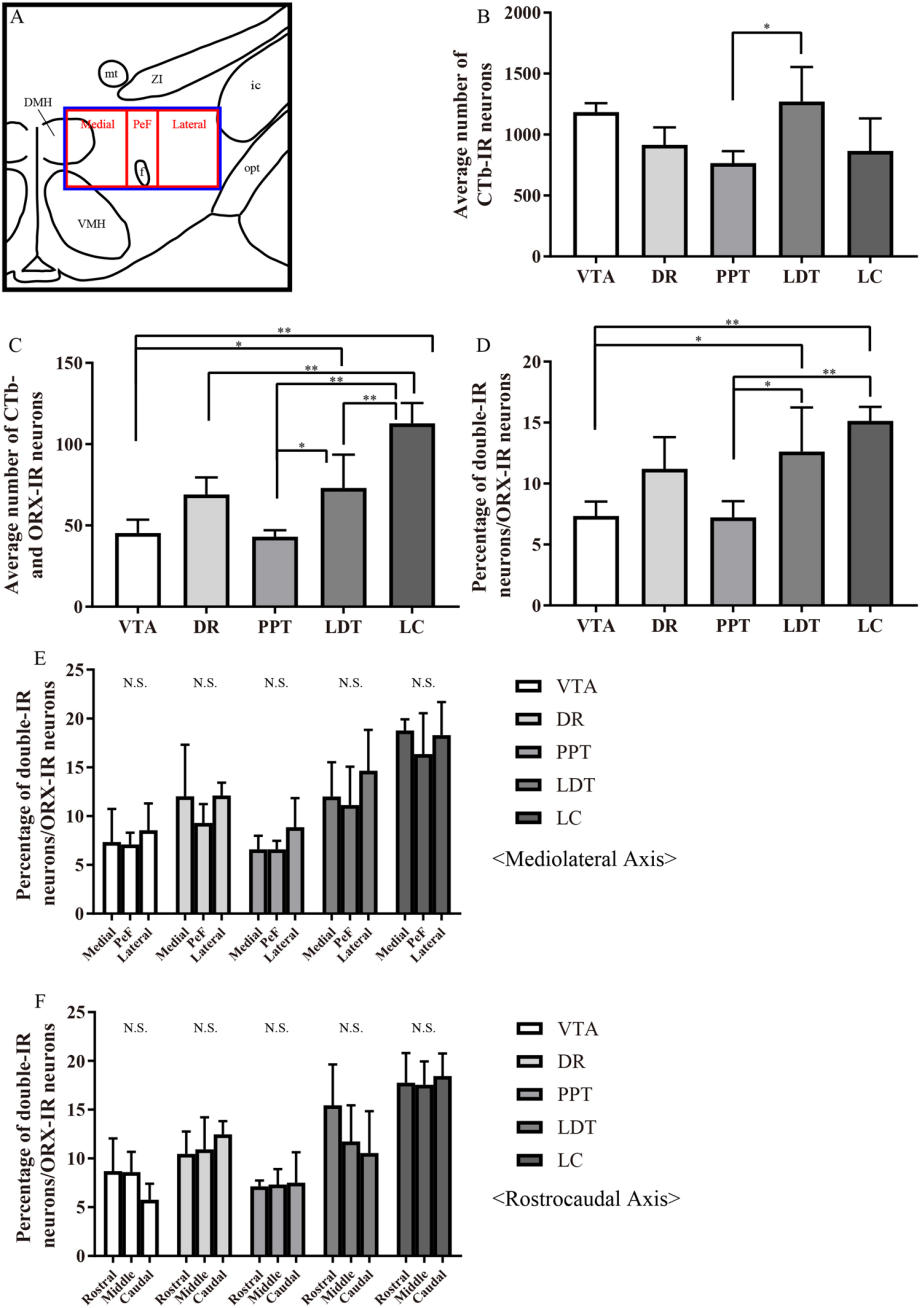
Abundant innervation of hypothalamic orexin neurons to LC. (**A**) Schematic drawing showing arbitrarily divided three regions of the hypothalamus. Blue square shows ORX field and this square is divided to three regions, medial, PeF, and lateral. (**B**–**D**) Quantification of the average number of CTb-labeled neurons (**B**), ORX-IR neurons and CTb-labeled neurons (**C**), and the percentage of ORX and CTb double-IR neurons in ORX-IR neurons (**D**) in the ORX field. *P < 0.05, **P < 0.01, (n = 4, 6 sections from each rat. one-way ANOVA followed by Tukey–Kramer test) (**E** and **F**) Quantification of the percentage of ORX and CTb double-IR neurons in ORX-IR neurons after an injection of CTb into the VTA, DR, PPT, LDT or LC. Cells were quantified in three rectangles, medial, PeF and lateral regions across mediolateral axis, and rostral, middle and caudal rectangles in the ORX field of the hypothalamus across the rostrocaudal axis and compared among samples. N.S.; not significantly different. PeF: perifornical area; CTb; cholera toxin b subunit; ORX: orexin; IR: immunoreactive; VTA: ventral tegmental area; DR: dorsal raphe; PPT: pedunculopontine nucleus; LDT: laterodorsal tegmental nucleus; LC: locus coeruleus.

We next examined the percentage of ORX-IR neurons labeled by CTb in the ORX field (Fig. 3A blue square). We counted ORX-IR neurons, CTb-labeled neurons, and ORX and CTb double-IR neurons in six sections from each rat (n = 4). We obtained confocal fluorescence microscopy images and analyzed the average number of ORX-IR neurons (Supplementary Fig. 8A), CTb-labeled neurons (Fig. 3B), and ORX and CTb double-IR neurons (Fig. 3C), and the percentage of double-IR neurons/ORX-IR neurons(Fig. 3D) or CTb-IR neurons(Supplementary Fig. 8B). There was no significant differences in the average number of ORX-IR neurons among all CTb injected regions (Supplementary Fig. 8A). When we injected CTb into the LDT, we detected significantly more CTb-IR neurons in the ORX field than after PPT injection (Fig. 3B). We found that ORX and CTb double-IR neurons were the most abundant after injection of CTb into the LC (Fig. 3C). In addition, when we injected into the LC, we observed a higher proportion of ORX-IR neurons that were double-labeled compared to the other regions (Fig. 3D). In this region we also observed a highest proportion of double-IR neurons/CTb-IR neurons (Supplementary Fig. 8B). Orexin neurons distribute unequally across mediolateral axis and rostrocaudal axis^20^ as shown in Supplementary Fig. 1. Therefore, to evaluate the mediolateral distribution pattern of ORX and CTb double-IR neurons in the ORX field, we divided the field into three regions (Fig. 3A red rectangles). In whichever region we injected CTb, ORX and CTb double-IR neurons were ubiquitously distributed in the medial, PeF, and lateral part of hypothalamus. There was no significant difference in neuronal distribution between the three areas as shown in Fig. 3E. Next, we divided the ORX field into three regions across the rostrocaudal axis, to examine the rostrocaudal distribution pattern of ORX and CTb double-IR neurons in the ORX field. In whichever region we injected CTb, ORX and CTb double-IR neurons were ubiquitously distributed in the rostral, middle, and caudal part of ORX field in the hypothalamus, as shown in Fig. 3F. These results suggested that more ORX neurons project to the LC rather than the VTA, DR, PPT or LDT. In addition, ORX neurons that do project to the VTA, DR, PPT, LDT and LC were ubiquitously distributed in the ORX field.

### Overlapping distribution of LPB neuronal fibers and ORX and CTb double-IR neurons projecting to the brainstem arousal areas

**Figure 4:**
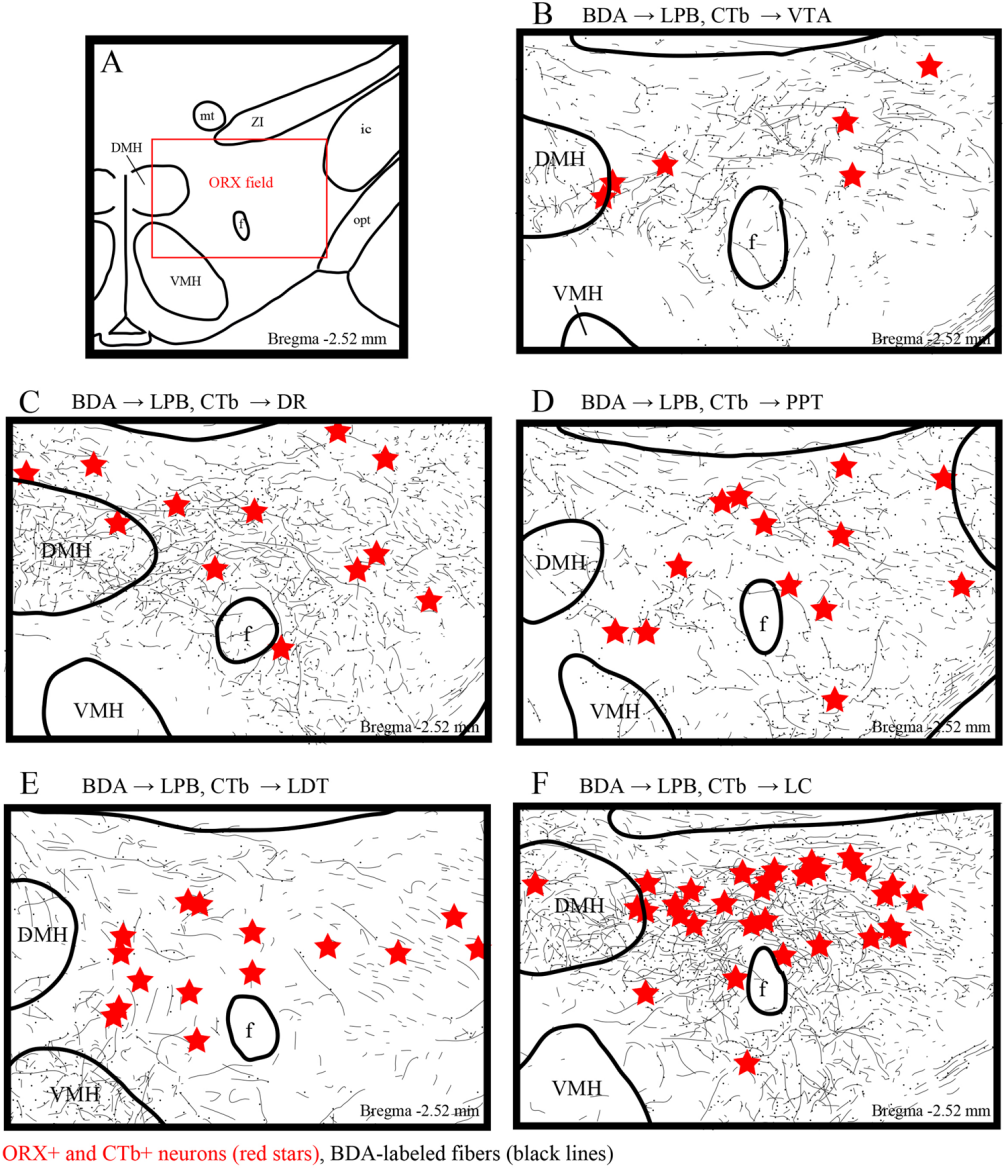
Distribution of LPB axons and ORX and CTb double-IR neurons in the ORX field. (**A**) Schematic drawing shows the ORX field. The distance (mm) behind the bregma is noted at the bottom right. (**B**–**F**) Line drawings showing the distribution of BDA-labeled axons (black line), and ORX and CTb double-IR neurons (red stars) in the hypothalamus. The distance (mm) behind the bregma is noted at the bottom right. LPB: lateral parabrachial nuclei; ORX: orexin; CTb: cholera toxin b subunit; BDA: biotinylated dextranamine.

Next, we analyzed the overlapping distribution of BDA-labeled axons and ORX and CTb double-IR neurons in the ORX field of the hypothalamus (Fig. 4A). When we injected BDA into the central lateral subnucleus of the LPB (Fig. 2B1,C1,D1,E1 and F1), we observed bilateral anterogradely labeled axons, but predominantly in the hypothalamus ipsilateral to the injected side. We also detected moderate to dense plexuses of labeled fibers in the DMH and adjacent areas lateral and dorsomedial to the nucleus (Fig. 4B–F, Supplementary Figs 3–7D–I). When we injected a small amount of extra BDA into the superior lateral subnucleus of the LPB (Fig. 2E1), anterogradely labeled axons also showed dense projection to the VMH (Fig. 4E and Supplementary Fig. 6D–I). In all cases, BDA-labeled fibers were distributed in the ORX field, across the tuberal hypothalamus. The most prominent overlapping distribution of BDA-labeled fibers and ORX and CTb double-IR neurons was found in the suprafornical area of the hypothalamus (Fig. 4B–F).

### Overlapping distribution of LPB axon terminals and brainstem arousal areas in the projections of ORX-IR neurons in the suprafornical area

**Figure 5:**
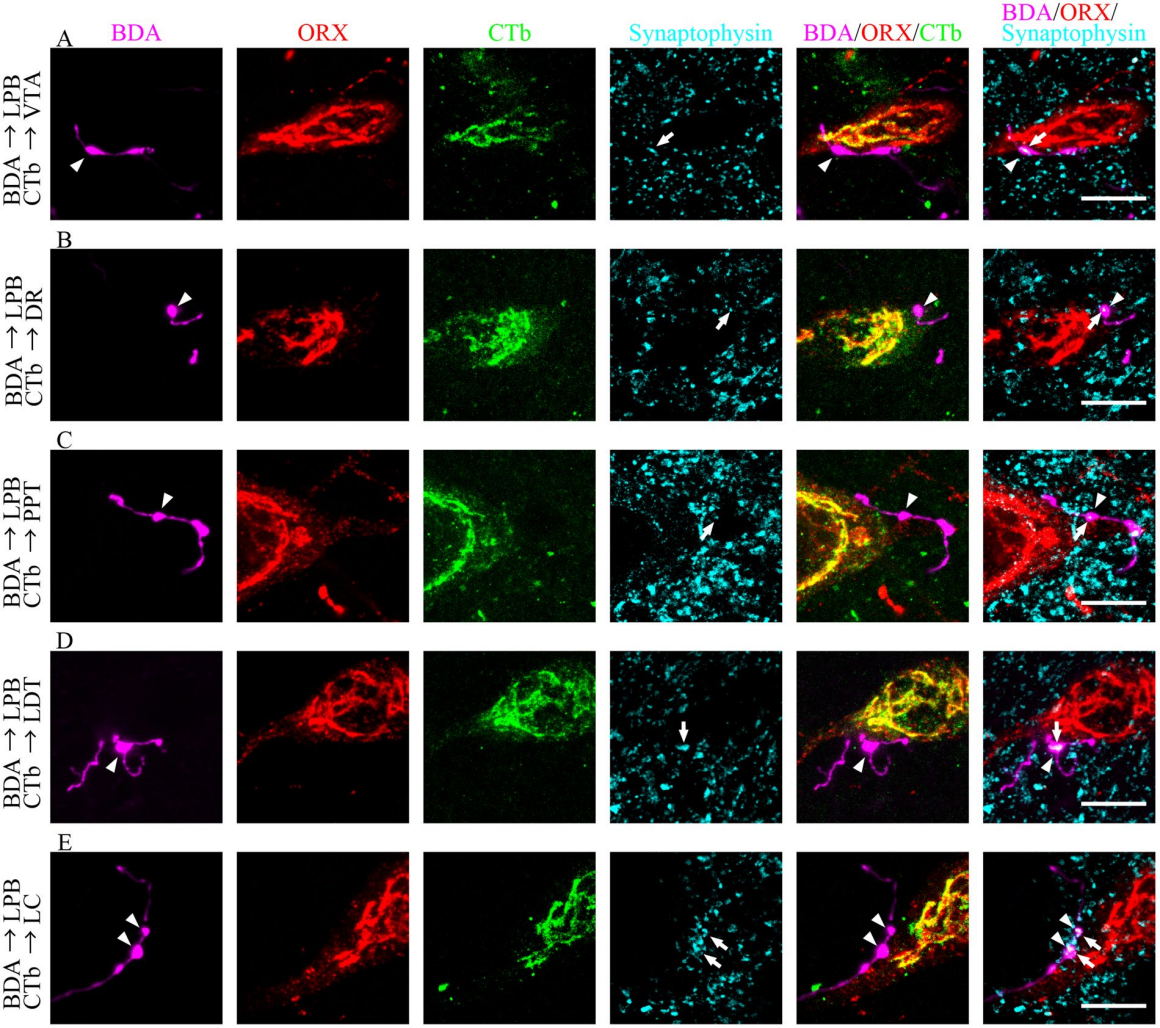
Confocal microscope images of quadruple fluorescence staining. Images show the appearance of CTb-labeled neurons (green) immunoreactive for ORX (red) and Synaptophysin (cyan) after injection of BDA (magenta) into the LPB and CTb into the VTA (**A**) DR (**B**) PPT (**C**) LDT (**D**) or LC (**E**) Two types of merged images, BDA/ORX/CTb and BDA/ORX/synaptophysin, were obtained from the same area. The arrowheads indicate BDA-labeled axons (magenta), which contact to ORX (red) and CTb (green) double-IR neurons. The arrows indicate synaptophysin (cyan) in BDA-labeled axons (magenta), which contact to ORX (red)-IR neurons. Scale bars, 10 μm. LPB: lateral parabrachial nuclei; ORX: orexin; VTA: ventral tegmental area; IR: immunoreactive; CTb: cholera toxin B subunit; BDA: biotinylated dextranamine; PPT: pedunculopontine tegmental nucleus; VTA: ventral tegmental area; DR: dorsal raphe; LC: locus coeruleus.

To examine whether axon terminals from LPB and ORX and CTb double-IR neurons were contiguous, we performed quadruple immunofluorescence staining and obtained confocal fluorescence microscope images (Fig. 5A–E). Enlarged ending of a BDA-labeled axon formed close contact sites with ORX-IR neurons with CTb immunoreactivity, suggesting the presence of synaptic contacts. To confirm whether these structures were indeed functional synapses, we examined the immunoreactivity for synaptophysin, a presynaptic marker, in those BDA-labeled axon terminals that directly apposed the VTA- (Fig. 5A), DR- (Fig. 5B), PPT- (Fig. 5C), LDT- (Fig. 5D), or LC-projecting (Fig. 5E) neurons. The results strongly indicated the existence of synaptic contacts at these locations.

## Discussion

In this study, we showed (1) prominent overlap of the distribution of LPB axons and ORX-IR neurons that project to the VTA, DR, PPT, LDT and LC in the rat; (2) that ORX neurons project to the LC more than to the VTA, PPT, LDT and DR; (3) that LPB axon terminals form synapses with ORX-IR neurons in the suprafornical area.

Our experiments provide an overview of overlapping distributions of LPB neuronal fibers and ORX-IR neurons projecting to wakefulness-regulating brainstem areas. We reproduced the previously demonstrated distribution pattern of ORX-IR neurons in the hypothalamus^1,3,5,6,21^ and also observed a similar distribution pattern of anterogradely labeled LPB^17^ neurons and retrogradely labeled neurons in the hypothalamus projecting to the VTA^22,23^, DR^24,25^, PPT/LDT^26,27^ and LC^28–30^ to those observed in previous studies.

How can we interpret the result that more ORX neurons projected to the LC than to other regions (Fig. 3C)? The LC is the major source of forebrain norepinephrine. LC neurons project widely throughout the central nervous system and receive inputs from other arousal systems, the brainstem, and the prefrontal cortex^30^. LC neurons promote arousal in general and are essential for the high levels of arousal required when responding to salient stimuli and stressors. LC activity is especially high under stress and in reaction to novel or salient stimuli that indicate reward or threat^31,32^.

The LPB receive nociceptive inputs from medullary (trigeminal) and spinal lamina I neurons^9,10^. The parabrachial nuclei constitute a main relay center for these inputs to areas of the forebrain, including the hypothalamus, amygdala, and bed nucleus of the stria terminalis^11–15^.

Putting these results together, the LPB-ORX-LC pathway could promote arousal via the projections to the LC when animals receive strong nociceptive inputs. However, several neuronal tracing studies reported reciprocal connections between the LPB and many of the other regions^8,33^ and some neurons in the LPB provide direct collateral projection to the VTA, DR, PPT, LDT and LC^34,35^ as we showed in the Supplementary Figs 9 and 10. These direct projections and newly identified indirect projections may form complex neural networks and modulate wakefulness. Further investigations using chemogenetic or optogenetical methods are needed to confirm how these specific neural pathways affect animals.

Significant numbers of ORX axons projects to areas other than the LC from the LPB, including the VTA, DR, PPT, and LDT. The VTA is the primary source of the mesocorticolimbic dopaminergic system. Dopamine is well known to drive arousal. Our findings suggest that signaling from the LPB could activate VTA dopamine neurons to promote wakefulness.

Most forebrain 5-HT arises from the DR and median raphe nucleus. These nuclei innervate and receive reciprocal inputs from many brain regions that regulate sleep/wake states, including the hypothalamus^36^. 5-HT neurons are sufficient to promote wakefulness, but these neurons are also implicated in regulating mood, reward, patience, and responding to salient cues. Our findings suggest that LPB ORX-IR neurons could not only promote wakefulness but also regulate mood and reward via projections to the DR.

The PPT and LDT are clusters of cholinergic neurons at the junction of the pons and midbrain^37^. PPT/LDT cholinergic neurons innervate many subcortical regions that influence arousal. Selective chemogenetic activation of PPT cholinergic neurons strongly suppresses slow electroencephalogram activity during non-REM sleep^38^, and photostimulation of these cells reduces slow wave activity during seizures^39^. Our data suggest that LPB ORX-IR neurons projecting to the PPT/LDT could influence cortical activity.

ORX neurons innervate a variety of brain regions, therefore each percentage of ORX neurons labeled with CTb injected to a particular area might seem to be low. However, the percentage of ORX neurons in the hypothalamus labelled by CTb injection to the LC, was reproducible (≈10–16%), as previously analyzed elsewhere^32^. As shown in Supplementary Fig. 8B, there is a possibility that other types of neurons could be involved rather than ORX neurons. As previously reported^40^, MCH or glutamatergic neurons^7^ could be a type of ORX-negative CTb -IR neurons.

With regard to the counting method, we adopted a manual counting method based on a paper that had been widely accepted in this field^41^. However, current researchers often take advantage of stereological techniques to estimate cell numbers. To justify our method, we compared the number of ORX-IR neurons in the hypothalamus obtained by both our manual counting method and the stereological method with Stereo Investigator software^42^. We counted hypothalamic ORX-IR neurons in every sixth section and obtained about 600 neurons in one side, as shown in Supplementary Fig. 8A. Therefore, we estimated ≈3600 neurons in one side of hypothalamus of male Wister rats. As examined in the previous study, using the same antibody or animals with the stereological technique^42^, they estimated ≈6700 in both sides of hypothalamus (≈3350/one side), which is definitely comparable with our results. Therefore, we believe that our counting method is as reliable as stereological technique.

This study has some technical limitations, such as variations in the spread of the tracers and the detection of synapses by synaptophysin immunoreactivity. Synaptophysin is a synaptic vesicle glycoprotein expressed exclusively at synapses^43^. The presence of synaptophysin was used to provide further evidence that the potential contact sites are actual synapses^44^. However, electron microscopy and electrophysiological experiments are needed to determine which of these appositions are true synapses and to examine inputs to distal dendrites. Additionally, the VTA, DR, PPT, LDT and LC displayed heterogeneity in their neuronal populations. Since we confirmed successful injection within these nuclei only approximately by immunohistochemical analysis against TH, ChAT and 5-HT, it is possible that the targeted neurons could be negative for these markers.

In conclusion, the present study showed the indirect LPB innervation via ORX neurons of the suprafornical area of the rat hypothalamus via projection fibers to the VTA, DR, PPT, LDT and LC in addition to direct pathways. More ORX neurons project to the LC rather than to the VTA, DR, PPT or LDT, suggesting that part of the arousal promoting of LPB is induced via the ORX-LC pathway.

These direct projections and newly identified indirect projections may form complex neural networks and control arousal. Further specific investigations are needed to confirm how these neural pathways affect wakefulness in animals.

## Materials and Methods

This study was approved by the institutional committee of Shimane University.

### Animals

All experiments were performed on male Wistar rats ranging in weight from 250 to 300 g. All surgical procedures were performed under general anesthesia, implemented by intraperitoneal injection of three anesthetic agents (0.3 mg/kg of medetomidine, 4.0 mg/kg of midazolam, and 5.0 mg/kg of butorphanol)^45^. All experiments were in compliance with the Guidelines for Animal Experimentation of the Center for Integrated Research in Science, Shimane University. The atlas of the rat brain from Paxinos and Watson (2005) was used to determine coordinates for stereotaxic injection of tracers as well as for the delineation of brain structures.

### Antibodies

See Supplementary Information (Supplementary Table 1).

### Combined anterograde tracing and retrograde tracing

Ipsilateral injections of BDA (Thermo Fisher Scientific, Waltham, MA, USA) into the LPB and CTb (List Biological Labs, Campbell, CA, USA) into the VTA (4 out of 10 rats received a successful injection), DR (4 out of 10 rats received a successful injection), PPT (4 out of 14 rats received a successful injection), LDT (4 out of 14 rats received a successful injection) or LC (4 out of 12 rats received a successful injection) were made stereotaxically by iontophoresis. In each rat, a single injection of CTb into these regions was made using a glass micropipette filled with 0.5% solution of CTb dissolved in 0.05 M phosphate buffer (PB; pH 6.0). The driving current (5–6 mA, 200 ms, 2 Hz) was delivered for 30–40 min. After CTb injection, a single injection of BDA was made into the LPB via a glass micropipette filled with a 10% solution of BDA dissolved in 0.01 M PB (pH 7.3). The driving current (5–6 mA, 200 ms, 2 Hz) was delivered for 40–60 min.

### Immunohistochemistry

Brain sectioning and immunohistotchemical analysis were performed as described previously^17,46^. Details are described in supplementary information.

### Illustration of BDA-labeled axons and ORX and CTb -IR neurons

For all illustrating studies, the images of immunofluorescent stained sections were collected using a confocal microscope. The montage images were acquired as multiple individual stacks using a 20x magnification in 3-step z-stacks through the full thickness of each sections. The images were stacked and tiled for enlarged views using Adobe Photoshop. After the images were arranged, we drew an illustration using the Adobe Illustrator software. BDA-labeled axons and IR cells were plotted on the layer above the tiled image.

### Analysis of ORX-IR neurons and CTb-IR neurons

We estimated what percentage of ORX-IR neurons were labeled by CTb in different parts of the ORX field such as the mediolateral axis and rostrocaudal axis. ORX-IR neurons, CTb-IR neurons, and ORX- and CTb-double IR neurons in each region were counted on six sections from each rat (n = 4 rats). The rostrocaudal axis of the ORX field was defined by bregma −2.28 mm to −3.48 mm, which is a region of densely distributed ORX neurons (Supplementary Fig. 1). For analysis of the mediolateral axis, we divided the ORX field into three regions according to methods described in previous studies^41,46^: a perifornical region defined by a 400 × 1000-mm rectangle centered on the fornix with the bottom edge just below the fornix; an 800 × 1000-mm medial region; and an 800 × 1000-mm lateral region (Fig. 3A). For analysis of the rostrocaudal axis, we divided the ORX field into three regions. A rostral and caudal part defined by the fist two sections and the last two sections, respectively. The middle part was defined by the two middle sections.

### Statistics

Data from ORX-IR neurons and CTb-IR neuron counts were compared using one-way ANOVAs, followed by Tukey–Kramer tests. All data are presented as mean ± SD. Statistical analysis was performed with GraphPad Prism 7 software (GraphPad Software, La Jolla, CA, USA).

## Supplementary information

Supplementary information

## Data Availability

All data generated or analyzed during this study are included in this published article and its Supplementary Information file.
