# Supplementary material for: Lateral parabrachial neurons innervate orexin neurons projecting to brainstem arousal areas in the rat
Source: Sci Rep. 2019 Feb 26;9:2830. doi: 10.1038/s41598-019-39063-y (PMC6391479; doi:10.1038/s41598-019-39063-y)
Supplement: Supplementary file 1 — Supplementary information [file 41598_2019_39063_MOESM1_ESM.pdf]

Supplementary information

Lateral parabrachial neurons innervate orexin neurons projecting to brainstem arousal areas in the rat

Yosuke Arima[1], Shigefumi Yokota[1], Masashi Fujitani[1]*

[1]Department of Anatomy and Neuroscience, Shimane University School of Medicine, 89-1 Enya-cho, Izumo-shi, Shimane 693-8501, Japan

*Corresponding author: Masashi Fujitani, Department of Anatomy and Neuroscience, Shimane University School of Medicine, 89-1 Enya-cho, Izumo-shi, Shimane 893-8501, Japan

Tel.:+81 0853 25 2106; Fax:+81 0853 20 2105

E-mail address: fujitani@med.shimane-u.ac.jp

Supplementary Table 1

Table 1 - Primary and secondary antibodies used for the present study.					
**Antigen**	Donor	Label	Dilution	Manufacture	Number
**Primary antibody**					
**ORX-A**	Rabbit	-	1:2000	Phoenix	H-003-30
**CTb**	Goat	-	1:5000	List Bio Lab	703
**CTb**	Rabbit	-	1:5000	Meridian	B65927R
**TH**	Mouse	-	1:2000	Immunostar	22941
**ChAT**	Goat	-	1:2000	Chemicon	AB144P
**5-HT**	Rabbit	-	1:2000	SIGMA	S 5545
**Synaptophysin**	Mouse	-	1:10000	SIGMA	S 5768
**Secondary antibody**					
**Goat IgG**	Donkey	biotin	1:500	Jackson	705-065-147
**Goat IgG**	Donkey	Alexa Fluor 405	1:1000	Abcam	ab175665
**Goat IgG**	Donkey	Alexa Fluor 488	1:1000	Invitrogen	A-11055
**Goat IgG**	Donkey	Cy3	1:1000	Jackson	705-165-003
**Goat IgG**	Rabbit	Peroxidase	1:5	NICHIREI	414181
**Mouse IgG**	Donkey	Alexa Fluor 488	1:1000	Invitrogen	R37114
**Rabbit IgG**	Donkey	Cy3	1:1000	Jackson	711-165-152
**Streptavidin**	-	Alexa Fluor 633	1:1000	Invitrogen	S21375
**Streptavidin**	-	Alexa Fluor 488	1:1000	Invitrogen	S11223
**DAPI**	-	-	1:1000	Dojin	D523

Supplementary method

Perfusion and brain sectioning

Five to seven days after injection, the rats were deeply anesthetized and perfused transcardially with 100 ml of saline, followed by 500 ml of 4% paraformaldehyde in 0.1 M PB (pH 7.3). The brains were removed, post-fixed in the same fixative for 3-4 h at room temperature and placed in a cold solution of 20% sucrose in 0.1 M PB. Subsequently, the brains were cut serially into frontal sections at 30-μm thickness on a cryotome. The sections were collected in 0.1 M PB. We obtained every sixth brain section from the whole serial sections for use in subsequent studies [1,2].

Immunohistochemical staining

Series of injection-site sections were washed in phosphate-buffered saline (PBS, pH 7.3), incubated in PBS containing 0.2% Triton X-100 for 3 h, and then incubated in PBS containing avidin-biotin-peroxidase complex (Elite ABC; Vector Labs, Burlingame, CA, USA) for 1 h. After washing in PBS, the sections were incubated in 25 ml of 0.1 M PB (pH 7.3) containing 10 mg diaminobenzidine (DAB), 5 mg nickel ammonium sulfate, and 10 ml 30% hydrogen peroxidase. BDA-labeled axons were stained dark blue to black. After washing in PBS, the sections were incubated in PBS containing 0.2% Triton X-100 and 3% normal rabbit serum for 1 h and then incubated overnight in PBS containing 0.2% Triton X-100, 3% normal rabbit serum, and goat anti-CTb antibody diluted at 1:5000. Subsequently, the sections were washed in PBS, incubated in PBS containing biotinylated

donkey anti-goat IgG secondary antibody diluted at 1:500 for 4 h, washed again in PBS, and then incubated in PBS containing Elite ABC for 1 h. After washing in PBS, the sections were incubated in 25 ml of 0.1 M PB (pH 7.3) containing 10 mg DAB and 10 ml 30% hydrogen peroxidase. CTb-labeled neurons were stained brown. After visual confirmation of staining, the sections were mounted onto gelatinized slides with Canada balsam (Wako Pure Chemical Industries, Ltd., Osaka, Japan), and imaged under a light microscope (Nikon Optiphoto-2; Nikon, Tokyo, Japan). Coverslips were then removed, and the sections were counterstained with 1% cresyl violet to better visualize cytoarchitectural landmarks[1,2].

Immunofluorescent staining

For all fluorescence immunostainings except for ChAT (described below), the sections were incubated in blocking buffer (PBS containing 0.3% Triton X-100 and 3% normal donkey serum) for 1 h and then incubated overnight in primary antibody with blocking buffer. Subsequently, the sections were washed in PBS and incubated in blocking buffer containing the secondary antibody for 4h. After washing in PBS, the sections were mounted onto gelatinized slides with ProLong® Diamond mounting medium (Thermo Fisher Scientific) and imaged with a BZ-X710 (Keyence, Osaka, Japan) for high-magnification images or a confocal microscope (Olympus FLUOVIEW FV1000; Olympus, Tokyo, Japan) for low-magnification images.

For ChAT staining, a Tyramide amplification procedure was used. Secondary antibodies consisted of anti-goat IgG peroxidase complex (1:5) in 0.01 M PBS for 1 h. After secondary antibody incubation, the samples were washed in PBS and then incubated for 5-10 min at room temperature in Tyramide-Rhodamine (1:1000) and glucose oxidase (1:1000). After this, D-glucose was added for 30 min and then the samples were washed in PBS. The imaging procedure follows that described above.

Coverslips were then removed, and the sections were counterstained with 1% cresyl violet for visualization of cytoarchitectural landmarks.

We arranged the pictures with Adobe Photoshop CC (Adobe Systems, Mountain View, CA, USA) and designed the line drawings and their labeling using Adobe Photoshop CC and Adobe Illustrator CC[1,2].

References

1 Niu, J. G., Yokota, S., Tsumori, T., Qin, Y. & Yasui, Y. Glutamatergic lateral parabrachial neurons innervate orexin-containing hypothalamic neurons in the rat. *Brain Res.* **1358**, 110–122 (2010).

2 Yokota, S., Oka, T., Asano, H. & Yasui, Y. Orexinergic fibers are in contact with Kolliker-Fuse nucleus neurons projecting to the respiration-related nuclei in the medulla oblongata and spinal cord of the rat. *Brain Res.* **1648**, 512–523 (2016).

Supplementary Figure 1

ORX/DAPI

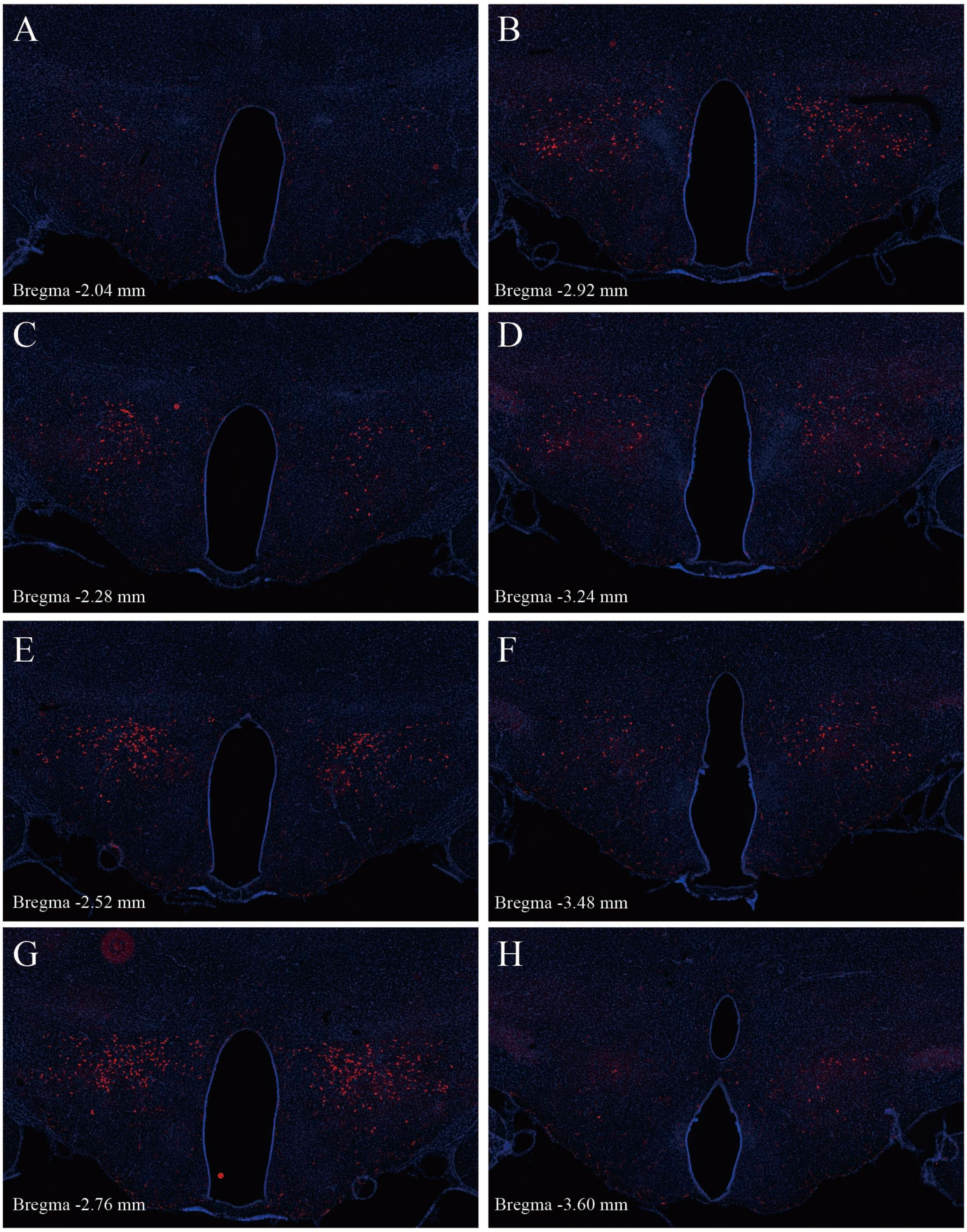

-/DAPI

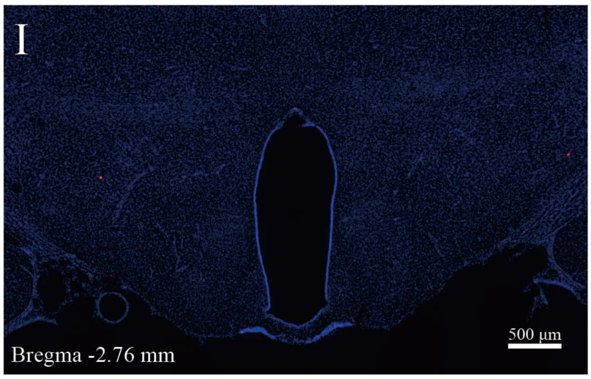

Supplementary Figure 1. Distribution of ORX-IR neurons in the hypothalamus.
The ORX/DAPI immunofluorescent staining images are shown in (A-H) from rostral(A) to caudal(H). no primary antibody control (I). Scale bar, 500 μm. The distance (mm) behind bregma is noted at the bottom in each images.

Supplementary Figure 2

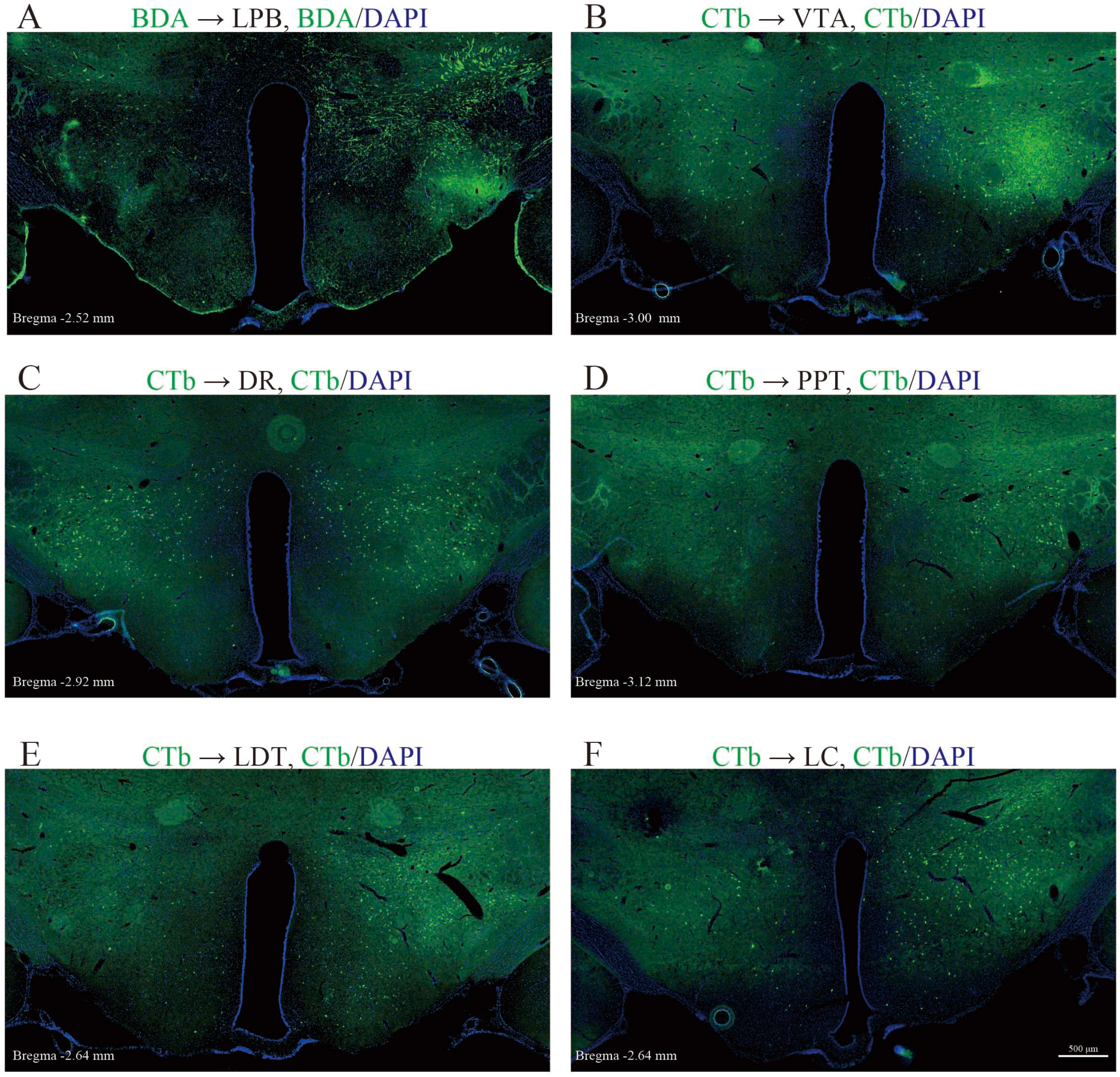

Supplementary Figure 2. Distribution of LPB fibers and VTA, DR, PPT, LDT and LC-projecting CTb-IR neurons in the hypothalamus.
The immunofluorescence images is shown in (A-F). (A) BDA injected into the LPB, BDA-labeled fibers distributed in the hypotalamus. The distribution pattern of CTb-labeled neurons are in the hypothalamus (B-F). CTb injected into the VTA (B), DR (C), PPT (D), LDT (E) and LC (F). in the hypothalamus. The distance (mm) behind bregma is noted at the bottom in each images. Scale bar = 500 μm.

Supplementary Figure 3

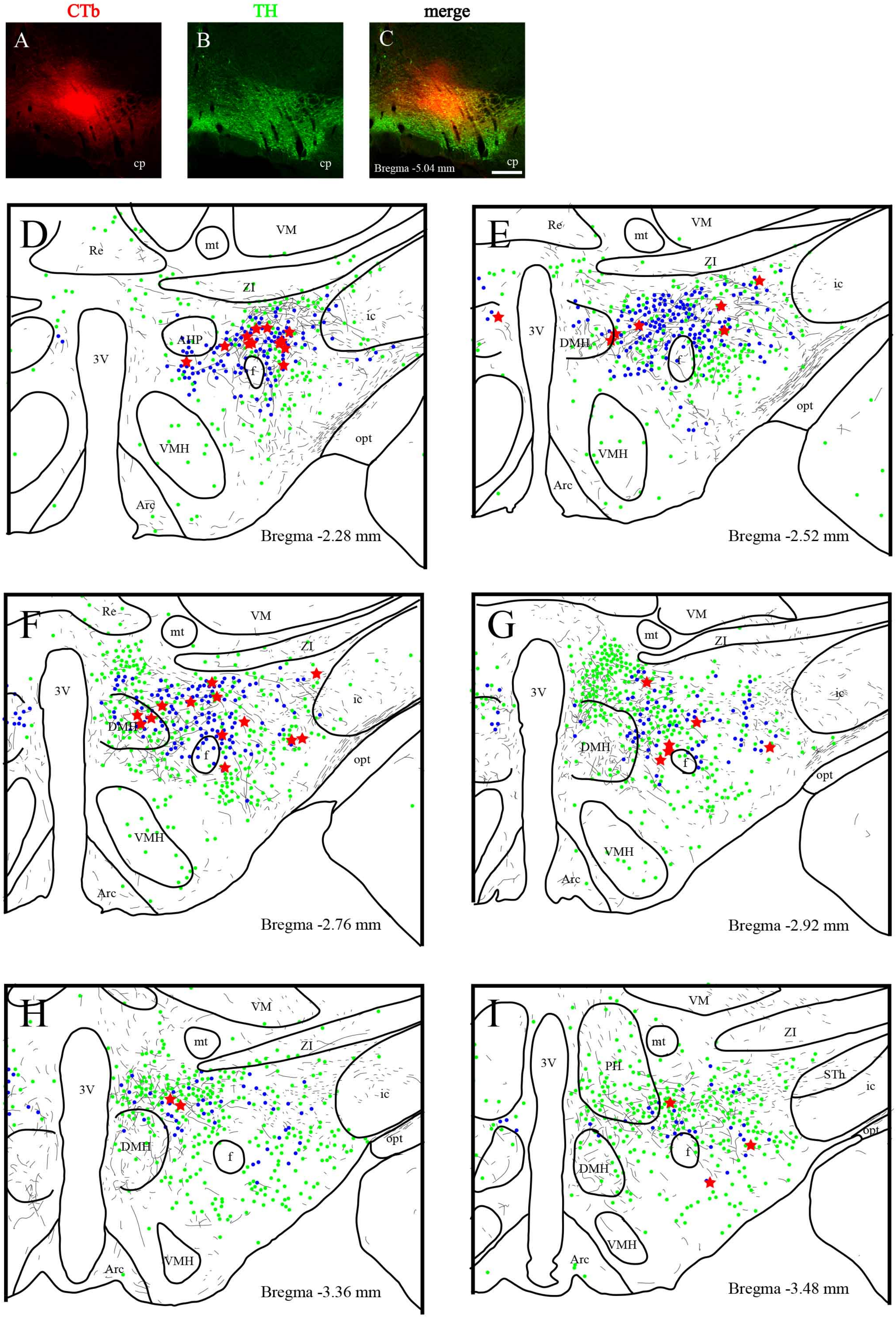

Supplementary Figure 3. Distribution of LPB axon terminals and VTA-projecting ORX-IR neurons in the hypothalamus. Confocal images showing the injection site of CTb into the VTA (red). (A) CTb injection site. (B) TH-IR (green) in the VTA. The merged image is shown in (C). Scale bar, 500 µm. (D-I) Line drawings showing the overlapping distribution of BDA-labeled fibers (black lines), CTb-labeled neurons (green filled circles), ORX-IR neurons (blue filled circles), and ORX- and CTb- double IR neurons (red stars) in the hypothalamus. The distance (mm) behind bregma is noted at the bottom in each drawing.

Supplementary Figure 4

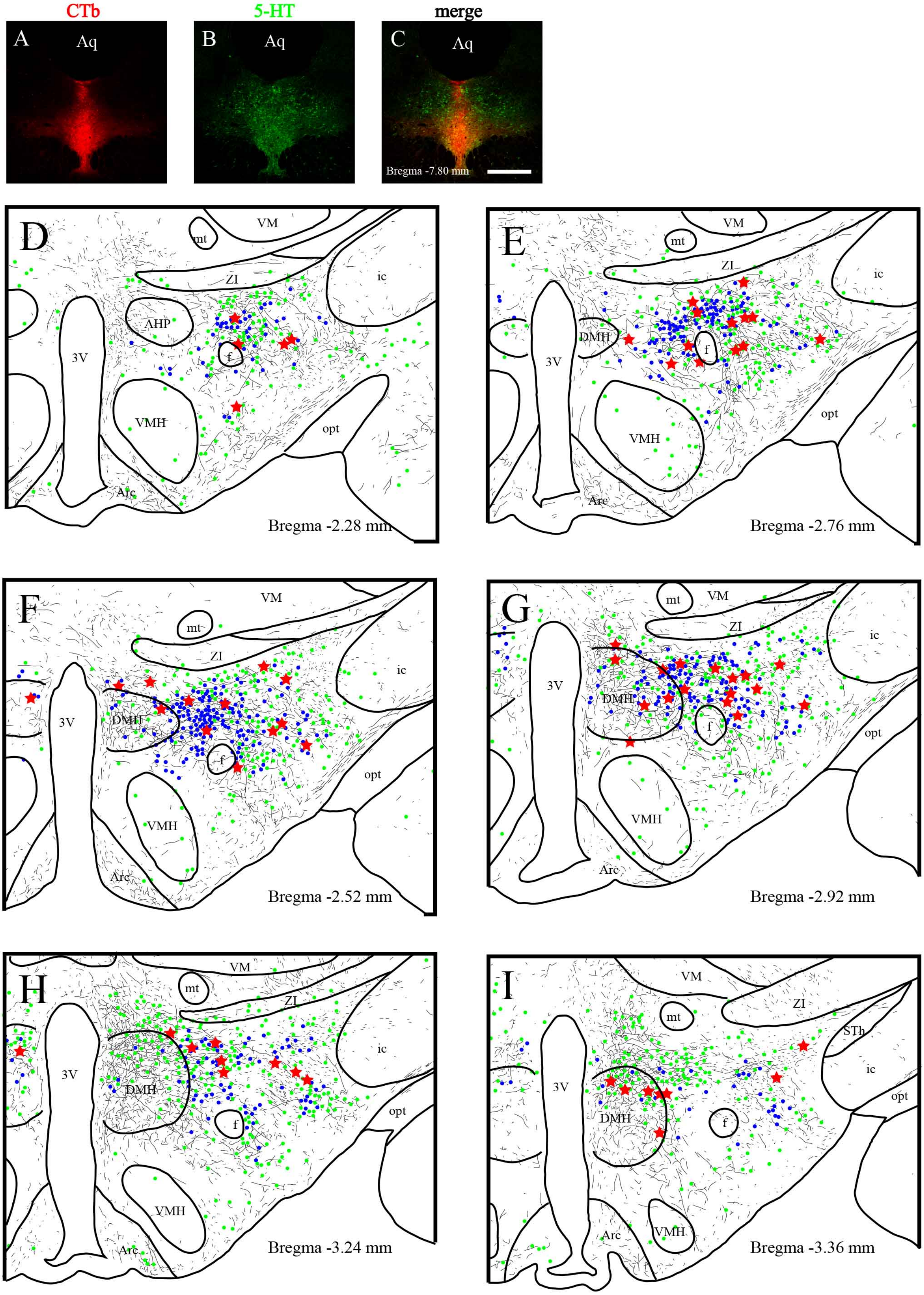

Supplementary Figure 4. Distribution of LPB axon terminals and DR-projecting ORX-IR neurons in the hypothalamus. Confocal images showing the injection site of CTb into the DR (red). (A) CTb injection site. (B) 5-HT-IR (green) in the DR. The merged image is shown in (C). Scale bar, 500 µm. (D-I) Line drawings showing the overlapping distribution of BDA-labeled fibers (black lines), CTb-labeled neurons (green filled circles), ORX-IR neurons (blue filled circles), and ORX- and CTb- double IR neurons (red stars) in the hypothalamus. The distance (mm) behind bregma is noted at the bottom in each drawing.

Supplementary Figure 5

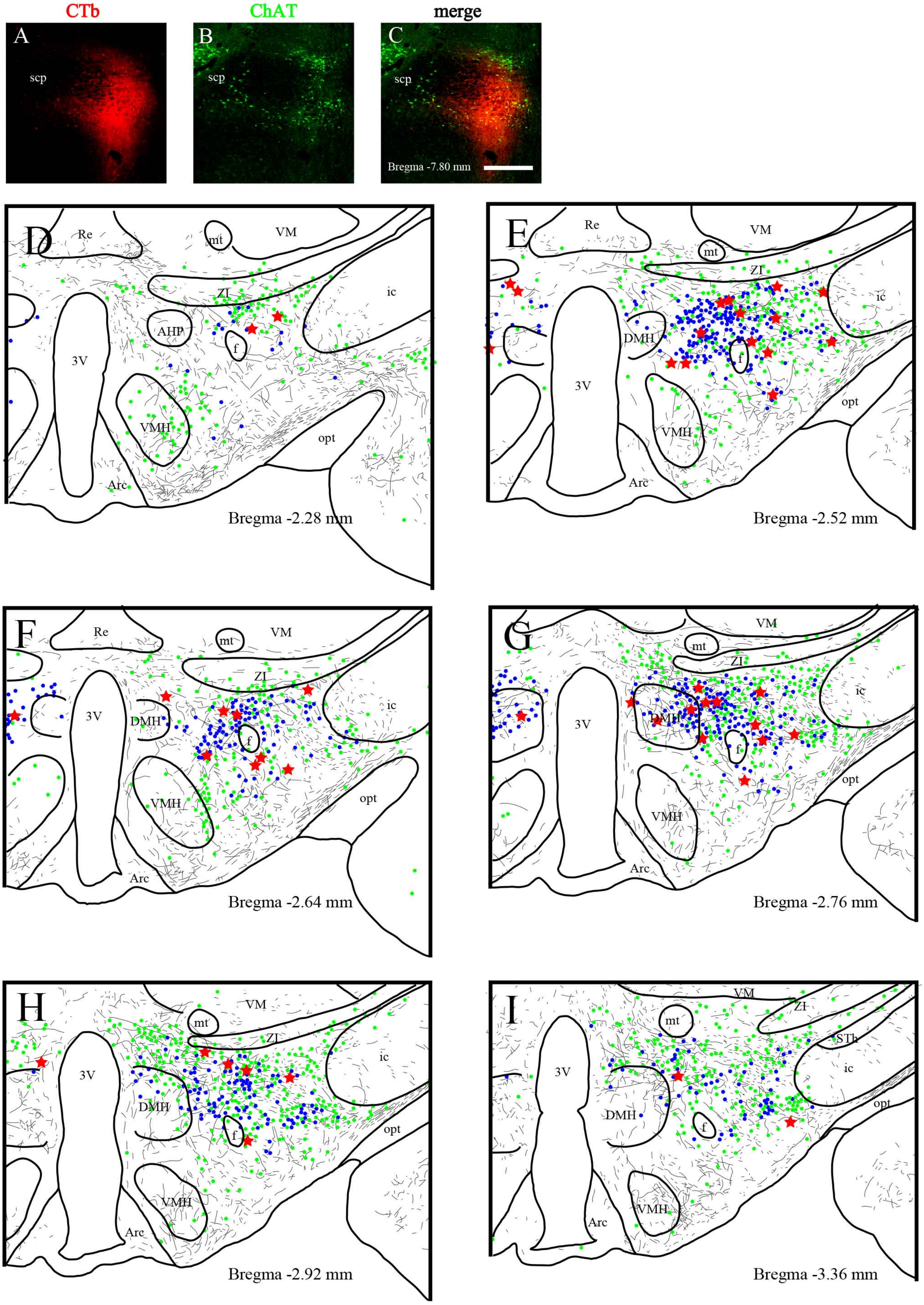

Supplementary Figure 5. Distribution of LPB axon terminals and PPT-projecting ORX-IR neurons in the hypothalamus. Confocal images showing the injection site of CTb into the PPT (red). (A) CTb injection site. (B) ChAT-IR (green) in the PPT. The merged image is shown in (C). Scale bar, 500 µm. (D-I) Line drawings showing the overlapping distribution of BDA-labeled fibers (black lines), CTb-labeled neurons (green filled circles), ORX-IR neurons (blue filled circles), and ORX- and CTb- double IR neurons (red stars) in the hypothalamus. The distance (mm) behind bregma is noted at the bottom in each drawing.

Supplementary Figure 6

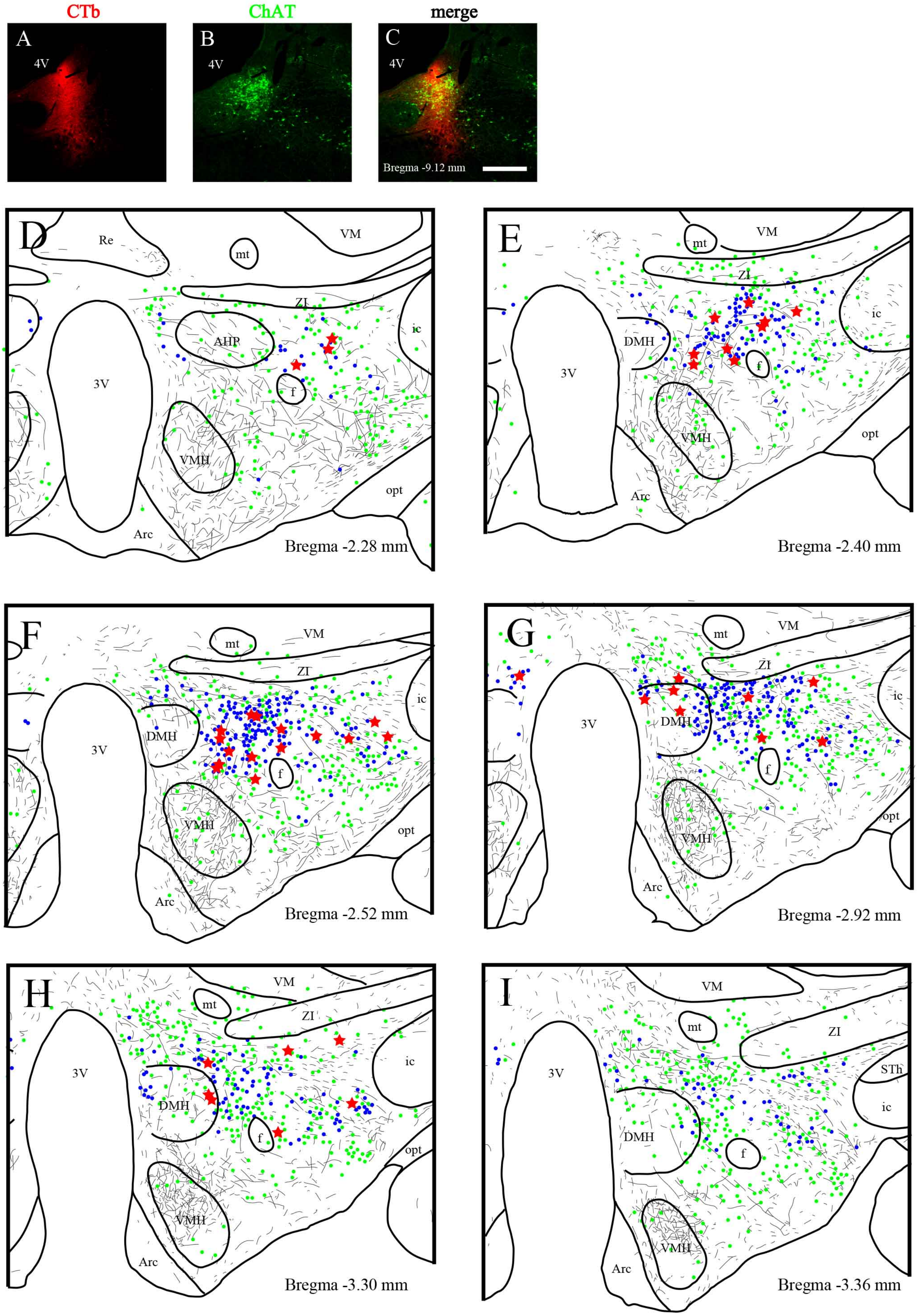

Supplementary Figure 6. Distribution of LPB axon terminals and LDT-projecting ORX-IR neurons in the hypothalamus. Confocal images showing the injection site of CTb into the LDT (red). (A) CTb injection site. (B) ChAT-IR (green) in the LDT. The merged image is shown in (C). Scale bar, 500 µm. (D-I) Line drawings showing the overlapping distribution of BDA-labeled fibers (black lines), CTb-labeled neurons (green filled circles), ORX-IR neurons (blue filled circles), and ORX- and CTb- double IR neurons (red stars) in the hypothalamus. The distance (mm) behind bregma is noted at the bottom in each drawing.

Supplementary Figure 7

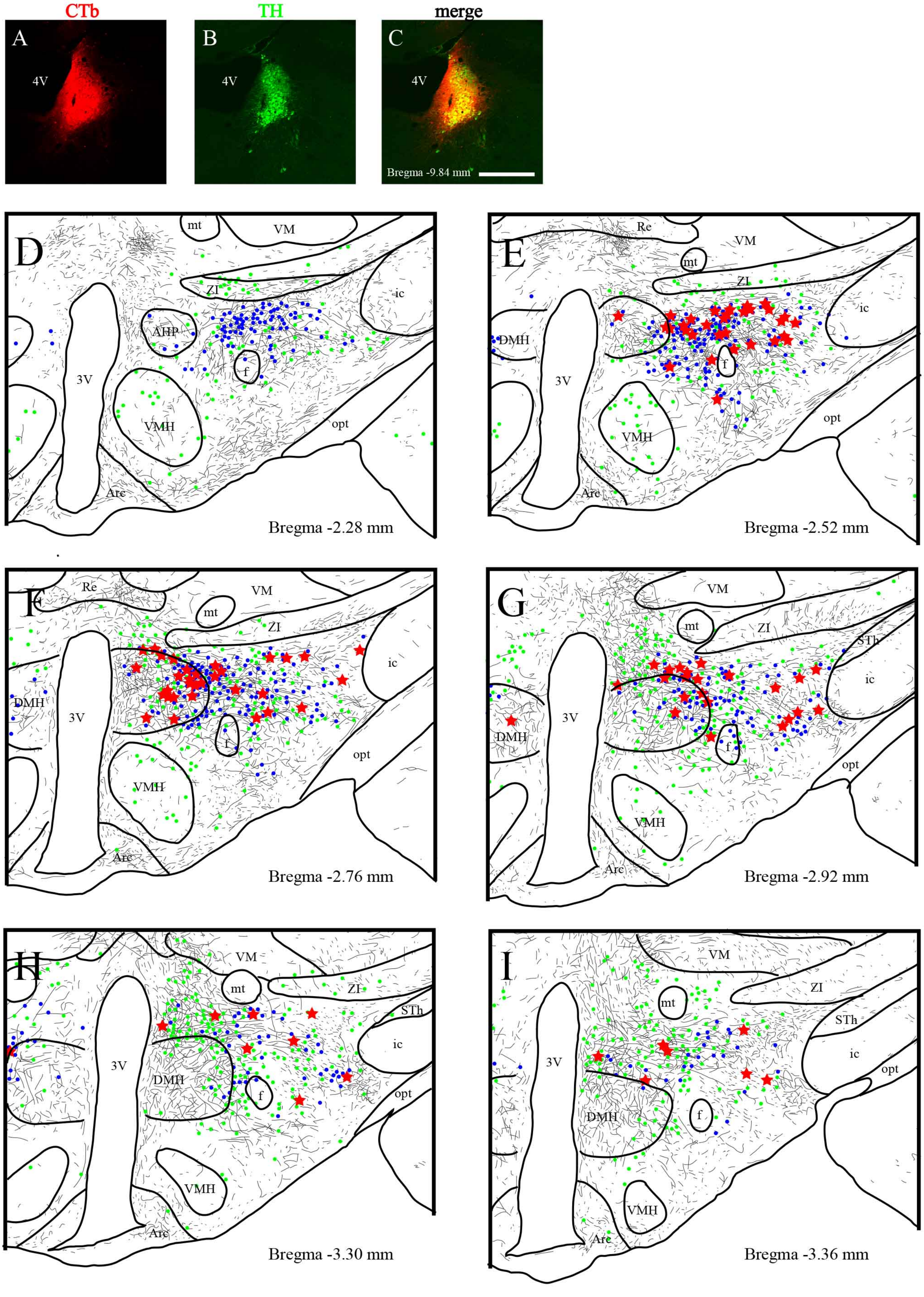

Supplementary Figure 7. Distribution of LPB axon terminals and LC-projecting ORX-IR neurons in the hypothalamus. Confocal images showing the injection site of CTb into the LC (red). (A) CTb injection site. (B) TH-IR (green) in the LC. The merged image is shown in (C). Scale bar, 500 µm. (D-I) Line drawings showing the overlapping distribution of BDA-labeled fibers (black lines), CTb-labeled neurons (green filled circles), ORX-IR neurons (blue filled circles), and ORX- and CTb- double IR neurons (red stars) in the hypothalamus. The distance (mm) behind bregma is noted at the bottom in each drawing.

Supplementary Figure 8

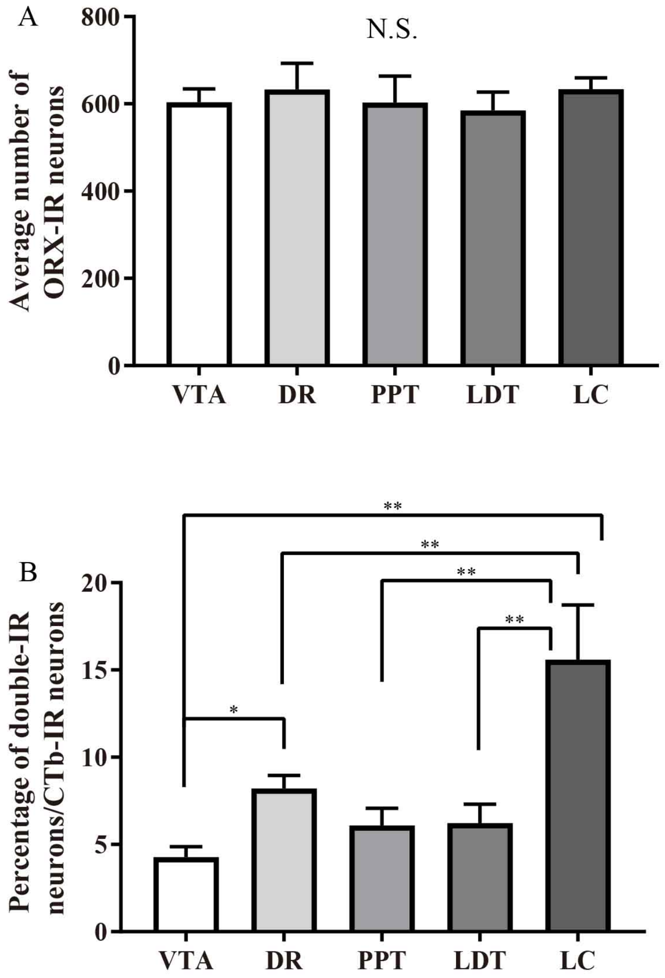

Supplementary Figure 8. Average number of ORX-IR neurons and percentage of double-IR neurons/CTb-IR neurons in the ORX field.

Quantification of the average number of ORX-IR neurons and percentage of double-IR neurons/CTb-IR neurons in the ORX field.

N.S.; not significantly different, **P* < 0.05, ***P* < 0.01, (n= 4, 6 sections from each rat. one-way ANOVA followed by Tukey–Kramer test)

Supplementary Figure 9

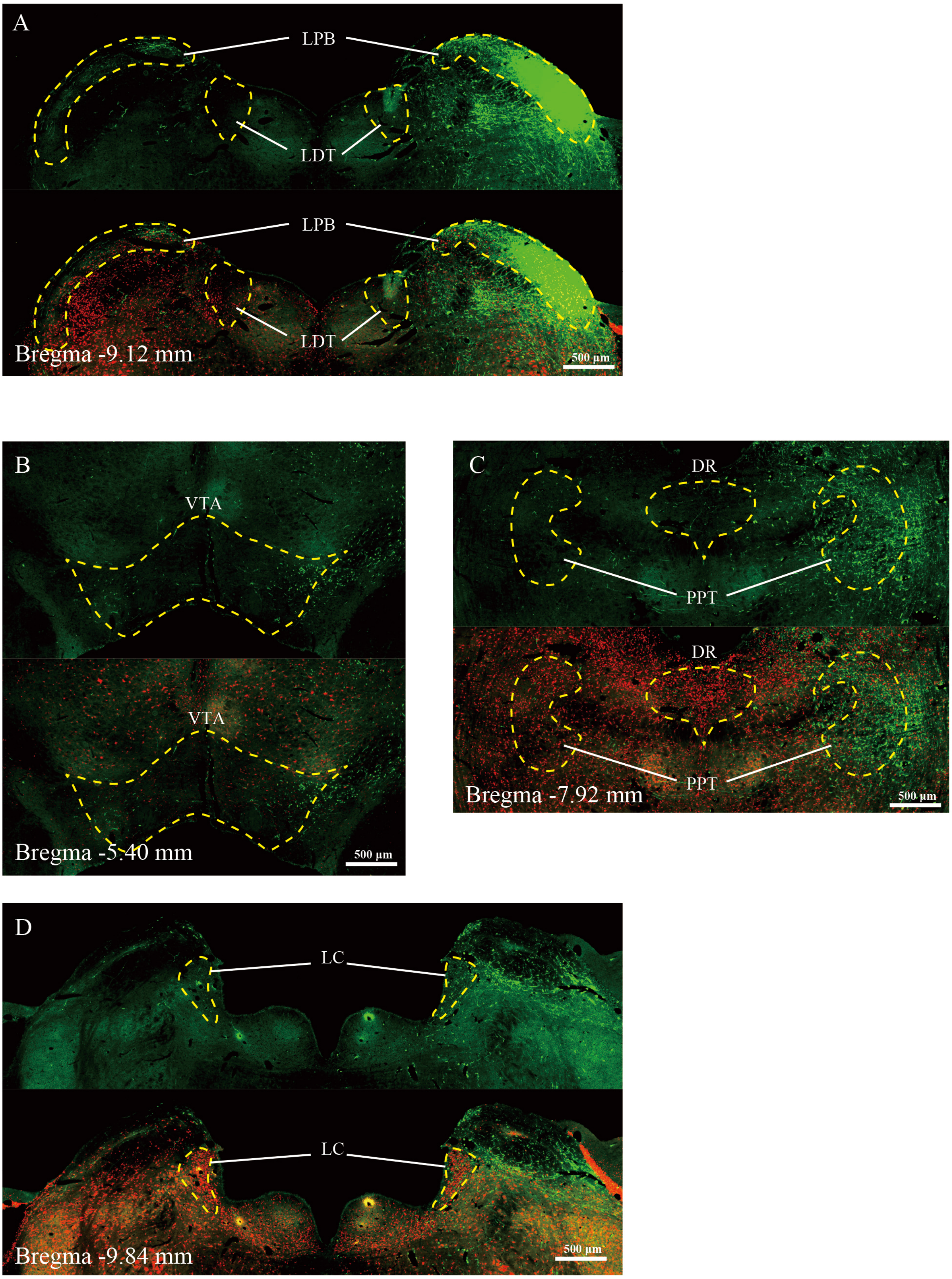

Supplementary Figure 9. LPB neurons project to VTA, DR, PPT, LDT and LC.
The BDA(green)/NeuN(red) immunofluorescent staining images are shown in (A-D). (A) BDA injection into the LPB (A) and the distribution of BDA-lebeled fibers in the LDT (A), VTA (B), DR (C) and (D).
Scale bar, 500 µm. The distance (mm) behind bregma is noted at the bottom in each images.

Supplementary Figure 10

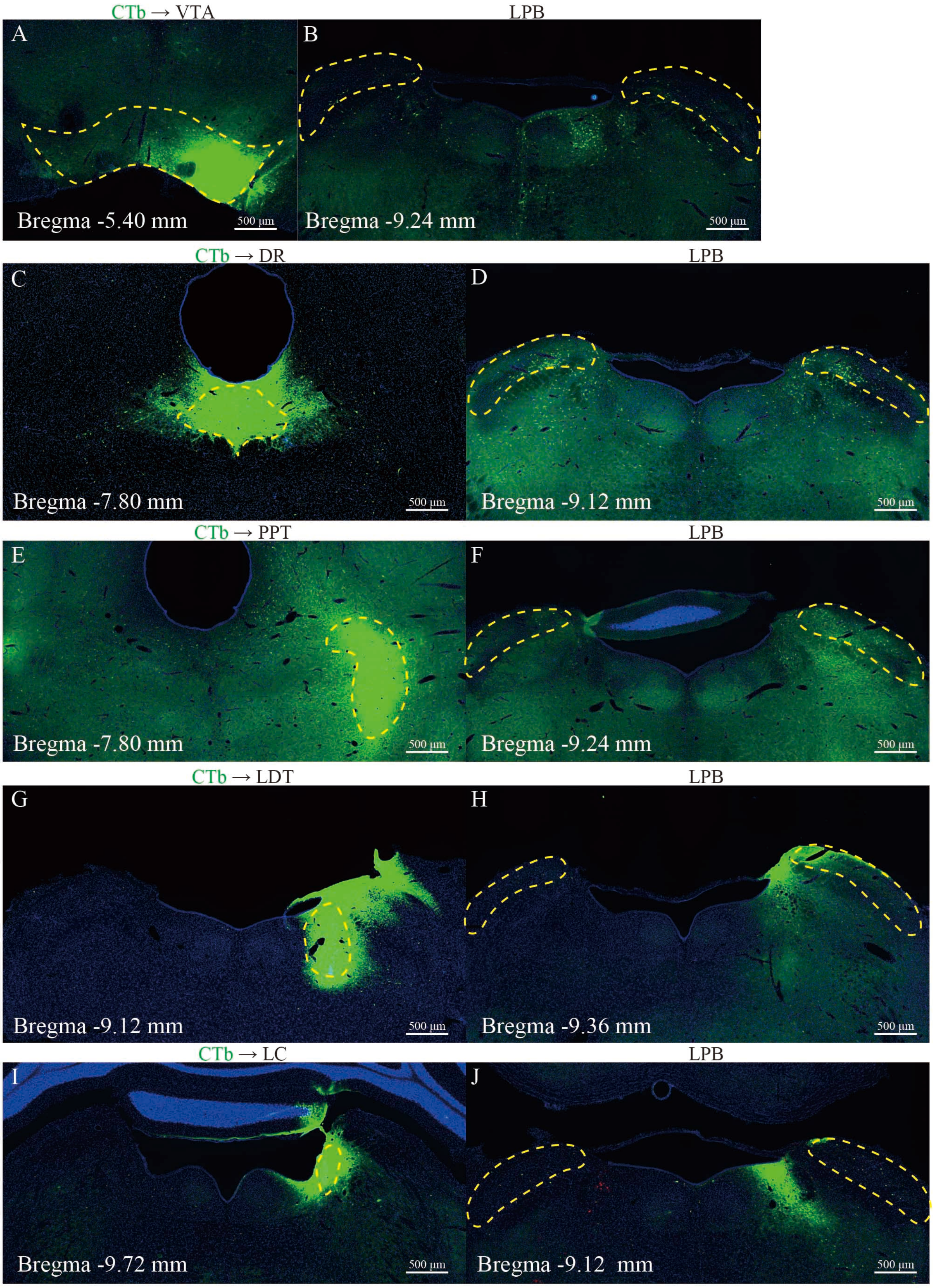

Supplementary Figure 10. Distribution of retrograde-labeled neurons in the LPB.
The CTb(green)/DAPI(blue) immunofluorescent staining images are shown in (A-J). CTb injection into the VTA (A), DR (C), PPT (E), LDT (G), and LC(I) and the distribution of CTb-labeled neurons in the LPB (B,D,F,H and J).
Scale bar, 500 µm. The distance (mm) behind bregma is noted at the bottom in each images.
